# Assessing the Probability that a Finding Is Genuine for Large-Scale Genetic Association Studies

**DOI:** 10.1371/journal.pone.0124107

**Published:** 2015-05-08

**Authors:** Chia-Ling Kuo, Olga A. Vsevolozhskaya, Dmitri V. Zaykin

**Affiliations:** 1 Department of Community Medicine and Health Care, University of Connecticut, Farmington, Connecticut, United Sates of America; 2 Department of Epidemiology and Biostatisitcs, Michigan State University, East Lansing, Michigan, United States of America; 3 National Institute of Environmental Health Sciences, National Institutes of Health, Research Triangle Park, North Carolina, United States of America; Pennsylvania State University, UNITED STATES

## Abstract

Genetic association studies routinely involve massive numbers of statistical tests accompanied by P-values. Whole genome sequencing technologies increased the potential number of tested variants to tens of millions. The more tests are performed, the smaller P-value is required to be deemed significant. However, a small P-value is not equivalent to small chances of a spurious finding and significance thresholds may fail to serve as efficient filters against false results. While the Bayesian approach can provide a direct assessment of the probability that a finding is spurious, its adoption in association studies has been slow, due in part to the ubiquity of P-values and the automated way they are, as a rule, produced by software packages. Attempts to design simple ways to convert an association P-value into the probability that a finding is spurious have been met with difficulties. The False Positive Report Probability (FPRP) method has gained increasing popularity. However, FPRP is not designed to estimate the probability for a particular finding, because it is defined for an entire region of hypothetical findings with P-values at least as small as the one observed for that finding. Here we propose a method that lets researchers extract probability that a finding is spurious directly from a P-value. Considering the counterpart of that probability, we term this method POFIG: the Probability that a Finding is Genuine. Our approach shares FPRP's simplicity, but gives a valid probability that a finding is spurious given a P-value. In addition to straightforward interpretation, POFIG has desirable statistical properties. The POFIG average across a set of tentative associations provides an estimated proportion of false discoveries in that set. POFIGs are easily combined across studies and are immune to multiple testing and selection bias. We illustrate an application of POFIG method via analysis of GWAS associations with Crohn's disease.

## 1 Introduction

Multiple statistical tests are routinely applied in genetic association studies and the corresponding P-values are reported. Journals require that P-values should be adjusted for multiple testing to protect against spurious findings. Nevertheless, findings often do not replicate in subsequent studies. Various explanations have been suggested for the low replicability of findings in observational studies, including inadequate accounting for multiple testing [1, 2]. In modern genetic studies, the number of statistical tests can be very large. Such discovery studies are often performed in a manner in which some small number of the most promising results are selected for closer investigation in a replication study.

It is now appreciated that a P-value does not reflect uncertainty about validity of a hypothesis. Yet P-values do contain information that can be used to evaluate this uncertainty, and we incorporate that information into our proposed method. A solution to the dilemma which findings are false and which are genuine can be obtained via conversion of P-values to Bayesian probabilities that a finding is genuine. A simple Bayesian solution has been proposed previously, termed the False Positive Report Probability (FPRP) [3]. In this approach, tailored to genetic association P-values, a plausible effect size, for example an odds ratio and the prior probability of the null hypothesis are proposed by a researcher, and “the probability of no true association” is determined for any finding with the P-value that is smaller than a preset threshold.

It has been suggested that the FPRP approach has two main deficiencies. First, as acknowledged by its authors, FPRP is not the probability that a *particular* finding is false, because it is based on the tail distributions rather than on the respective densities. The FPRP approach advocated plugging in an observed P-value in place of a fixed threshold. The result can only be interpreted as “the lowest FPRP for which the finding meets a preset criterion for noteworthiness” [4]. In his critique of the FPRP, Lucke [5] wrote that “the FPRP can promote false positive results”, due to a peculiar property of the FPRP that it cannot exceed the proposed prior probability. Secondly, the usage of a single “typical” value of the odds ratio fails to acknowledge that in reality different genuine signals carry different effect sizes and a proper calculation should take into account the entire *distribution* of possible effect sizes. Lucke was not optimistic regarding performance of methods such as FPRP built using this simplification [5]. However, the extent of imprecision introduced by the usage of a single value remains unclear. Lastly, we note that in the FPRP approach, all variants are divided into two classes, the first class containing those that are truly associated, and the second class containing variants with the effect size that is precisely equal to zero. The second class is assumed to contain majority of the variants and corresponds to the sharp null hypothesis, *H*_0_. While such approach is common due to its convenience, the non-associated set is likely to contain many variants with effect sizes sufficiently small to be either inconsequential to a researcher or undetectable given a particular sample size. It is more realistic to define the null hypothesis in such a way as to allow inclusion of variants with negligibly small effects.

The FPRP method is simple to apply and it has been referenced extensively since its introduction in 2004. Similarly simple methods are needed to convert a particular P-value to the “probability of no true association”, so that the result can be interpreted as the probability about this particular report. One such method has been proposed by Wakefield, called the “Bayesian false-discovery probability” (BFDP) [6]. In BFDP, the prior distribution of effect sizes, specified in terms of the log relative risk, is assumed to be normal. Strength of association is modeled via the variance of that distribution. BFDP has a sound Bayesian foundation, but the focus on the relative risk and the usage of the normal prior distribution for the effect sizes might be somewhat restrictive.

**Fig 1:**
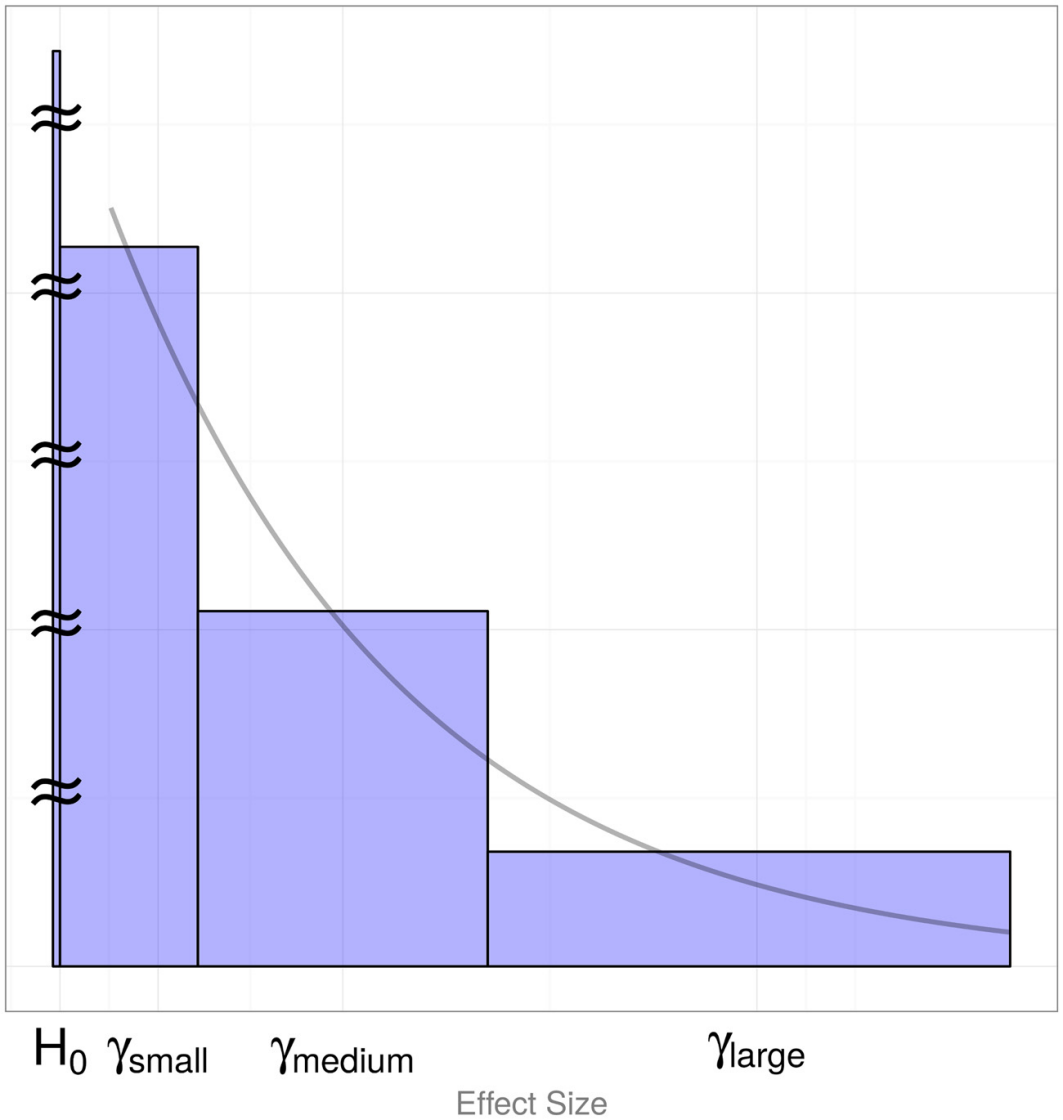
Approximating the effect size distribution by three values. Discretization of the Gamma(shape = 1, scale = 1) distribution. The middle bin is centered around *γ*_medium_ = *μ* = scale×shape = 1 and has the width *μ*.

Here we introduce POFIG, “Probability that a Finding is Genuine”—a solution inspired by the idea behind the FPRP approach. Our method shares generality and simplicity of FPRP in that it utilizes P-values that may result from various association statistics. POFIG alleviates the main shortcomings identified with the FPRP approach. The usage of a single value taken to be the mean of the underlying effect size distribution does introduce a bias that can be substantial for very small P-values. However, a modification based on just three typical values (small, medium, and large) with their respective frequencies provides a much better approximation to the complete specification of the effect size distribution. Typically, most signals would carry small effect sizes, some signals would fall into the middle group around the mean of the effect size distribution, and signals of large magnitude would represent only a small proportion of the distribution. In addition, the “null bin” can also be defined to capture magnitudes of effect sizes that are close to zero (Fig 1). Instead of testing the usual null hypothesis that a variant has precisely zero effect, one can test a hypothesis whether the effect associated with a variant is sufficiently close to zero, i.e., falls into the null bin. Statistical methods are starting to appear that allow to characterize the effect size distribution in a tabulated way. Park et al. proposed a method to extract tabulated effect size values with their abundances from genome-wide association studies of complex traits [7]. Our method is designed to utilize such tables of estimated or plausible effect sizes. Correct specification of the effect size distribution becomes especially important when top hits are selected from a study with very many tests. When the distribution of effect sizes is specified correctly, posterior probabilities of association are completely unaffected by multiple testing and by the process of selection of the most significant results. In an experiment with multiple tests, one can sort results by an association statistic and take the most extreme one: the POFIG calculations do not depend on the number of tests. POFIG is similarly unaffected by the process where only those multiple testing experiments are retained which contained statistically significant results. Computed probabilities that a finding is genuine are still correct when selectively applied to experiments that contained statistically significant findings.

## 2 Results

Our method estimates the posterior probability of the null hypothesis (*H*_0_) that an association is spurious, or the posterior probability of the alternative hypothesis (*H*_*A*_) that an association is genuine. These probabilities are estimated given the association strength summarized by a P-value (*p*). POFIG can be defined using the Bayes rule in a traditional way as POFIG = Pr(*H*_*A*_|*p*) = 1 − Pr(*H*_0_|*p*),

$$\begin{array}{c} \text{POFIG}=1-{\left[1+\frac{1-\mathrm{Pr}(H_{0})}{\mathrm{Pr}(H_{0})}\times f_{\bar{\gamma }}\left(p\right)\right]}^{-1}. \end{array}$$ (1)

POFIG depends on the marginal probability density of P-values for truly associated variants, given by

$$\begin{array}{c} f_{\bar{\gamma }}\left(p\right)=\frac{\sum_{i=1}^{B}w_{i}f_{\gamma_{i}}\left(p|\gamma \right)}{\sum_{i=1}^{B}w_{i}}. \end{array}$$ (2)

In calculating this marginal density one specifies *B* plausible ranges (i.e, “bins”) of effect sizes, with the effect size value for the bin *i* given by *γ*_*i*_, and the number of loci with that effect size (*w*_*i*_). The index *i* = 1, …, *B* tracks the current bin in the summation. If the total number of loci in the genome is *K*, the prior probability of the null hypothesis is Pr(*H*_0_) = 1 − ∑*w*_*i*_/*K*.

Consistent with the FPRP and BFDP approaches, the above formulation presents the null hypothesis as sharp, i.e, as a special class for signals with precisely zero effect size. In an alternative specification of POFIG, the null hypotheses can be defined to encompass variants with effect sizes that are small enough to be considered undetectable. This formulation has a number of advantages. The sharp null hypothesis, *H*_0_:*γ* = 0 is a convenient statistical concept, however it implies that the effect size distribution has a spike at a single point *γ* = 0, which is biologically unrealistic. Instead, it is believed that there is a very large number of variants with tiny effect sizes that influence common traits and complex diseases. These effects are of negligible size when a particular variant is considered by itself, but collectively these effects may explain a substantial proportion of heritability [8, 9]. Rather than treating null effects as a special class, we define an extra bin for the effects with the average effect size equal to *γ*_0_, in a narrow interval near zero and the number (or the proportion) of such effects, *w*_0_. The posterior probability of the null hypothesis is evaluated via

$$\begin{array}{ccc} \mathrm{Pr}(H_{j}|p) & = & \frac{\mathrm{Pr}\left(H_{j}\right)f_{\gamma_{j}}\left(p|\gamma \right)}{\sum_{i=0}^{B}\mathrm{Pr}\left(H_{i}\right)f_{\gamma_{i}}\left(p|\gamma \right)} \end{array}$$ (3)

with *j* = 0. The posterior probabilities for the rest of the bins (i.e., *j* = small, medium, large) can be calculated similarly. The weights *w*_*i*_ in Eq (2) are proportional to Pr(*H*_*i*_) and $\sum_{i=0}^{B}\mathrm{Pr}(H_{i})=1$. The posterior mean for the effect size

$$\begin{array}{c} E(\gamma |p)=\sum_{i=0}^{B}\gamma_{i}\;\mathrm{Pr}\left(H_{i}|p\right)\;. \end{array}$$ (4)

The summation index in Eqs (3, 4) now starts from zero rather than from one to accommodate inclusion of the null bin that is no longer associated with the point null hypothesis (*H*_0_), as in Eq (1). The effect size in Eqs (1–4) is defined as a parameter of the P-value density. As an example, we will use the usual one degree of freedom chi-square SNP association statistic, but the P-value density is defined in the same way for statistics that have distributions other than the chi-square (details are given in Materials and Methods). The P-value density is

$$\begin{array}{ccc} f_{\gamma_{i}}\left(p|\gamma \right) & = & \frac{g_{\gamma_{i}}(G_{0}^{-1}\left(1-p\right))}{g_{0}(G_{0}^{-1}\left(1-p\right))}, \end{array}$$ (5)

where *g*_*γ*_*i*__(·) is the one degree of freedom noncentral chi-square density function with the noncentrality *γ*_*i*_ and subscript 0 refers to the central chi-square density. The function $G_{0}^{-1}(\cdot )$ is the inverse central chi-square cumulative distribution with one degree of freedom. Tabulated effect size values can be given in terms of the odds ratios and then related to noncentralities, assuming a logistic regression model:

γ=N×Dq(1-q)=N×𝓔, (6)

where *D* is the squared log of odds ratio, *N* is one half of the harmonic mean of case and control sample sizes (*n*_1_, *n*_2_, in allele counts), *N* = *n*_1_ *n*_2_/(*n*_1_ + *n*_2_), and *q* is the pooled allele frequency. Thus, the effect of an increase in sample size is an increase in the noncentrality and an increased skewness toward zero in the distribution of P-values. ℰ can be interpreted as a standardized effect size: 2ℰ corresponds to the contribution of a SNP to the additive genetic variance of the trait. Park et al. gave a method for estimating the distribution of ℰ that utilizes data from replicated GWAS findings [7]. As noted above, the effect size distribution can be modeled in terms of parameters, such as relative risk or odds ratio, or directly in terms of the noncentralities. Park et al. used a standardized effect distribution which is the same as the distribution defined in terms of noncentralities. When sample sizes are the same for all tested variants in a study, placing the distribution directly on ℰ (equivalently, on *γ*) gives posterior probabilities that have the same ranks as P-values. On the other hand, specification of the distribution for *D* will give probabilities with ranks that differ from the ranks of P-values. In SNP association testing, the same noncentrality (*γ*) may result from a SNP with a large odds ratio and a small allele frequency, as well as from a SNP with a small odds ratio but a large allele frequency. Thus, usage of the standardized effect size implicitly assumes that rare variants tend to have a larger effect size compared to common variants.

Our approach utilizes a weighted sum of *B* tabulated effect size values (Eqs 1–3). At one extreme we may consider a single “typical” effect size value (*B* = 1), as in the FPRP approach. At the other extreme, the effect size distribution may be represented by a continuous function. These modifications and the relation between POFIG and FPRP are given in Materials and Methods.

Representation of the effect size distribution by a discrete set of bins offers several advantages. Among them are (1) simplicity of calculations; (2) emergence of methods for estimation of effect size distribution in human genome [7]; (3) straightforward specification by practitioners, e.g., it is easier for a researcher to come up with several plausible effect sizes and their relative abundances than to specify parameters of a continuous distribution; (4) flexibility of the approach: the binned distribution does not need to be cast in a form of a parametric statistical distribution, such as a normal distribution, and thus can reflect more closely a biologically realistic distribution.

The advantages of our approach might be offset if the number of bins (*B*) is too small to represent the actual effect size distribution for the purpose of estimating posterior probabilities with good precision. It is especially important that the probabilities are well-estimated for top hits of a study, i.e., for those variants that are selected based on the smallest association P-values. Such selection of top hits is known to induce bias known as the “winner’s curse” phenomenon, in which selected P-values tend to be too small and the corresponding estimated effect sizes tend to be too large. Correcting this bias is known to be difficult [10]. Fully Bayesian inference is not susceptible to the winner’s curse [11], as long as the prior distributions are specified correctly. In our case, prior for the effect size distribution needs to reflect the actual prevalence of all possible signal magnitudes in the genome. Our proposal to collapse the effect size distribution into discrete bins may induce imprecision and make the method susceptible to the winner’s curse bias. However, our results demonstrate that even a crude approximation based on three ranges for effect sizes provides good protection against bias due to selection of top strongest signals in a study.

### 2.1 Simulation results

Simulation experiments were designed for judging precision of our method in evaluating the proportion of true signals among smallest P-values in multiple-testing experiments. We assumed that the effect size is represented by the odds ratios (OR) and that the squared logarithm of OR has an L-shaped distribution. For any true effect, its effect size was sampled from an L-shaped distribution and the corresponding P-value was obtained based on that effect size value. We also allowed for a proportion Pr(*H*_0_) of signals to be completely spurious (false), i.e., to have the OR of exactly one. In a single multiple-testing simulation experiment with *K* P-values, each P-value carried a flag indicating whether it originated from a true (T) or a false (F) signal. Once *K* P-values were sorted, we examined the fraction of flags that were equal to T in a set of *K* smallest P-values. This gives an empirical (i.e., true) proportion of true signals among *K* smallest P-values for a single simulation experiment. Our POFIG method provides an estimate of this proportion: it is the average of POFIG values for these *K* smallest P-values. Next, we averaged the true (empirical) and the POFIG-estimated proportions over a large number of simulation experiments. Given this simulation setup, correctness of our method can be judged by closeness of the estimated values to the true values obtained using the knowledge of which P-values are actually generated from true signals. This gives an overview of the simulation setup; more detailed description is given in Material and Methods.

In the simulation experiments, we defined the effect size *D* in terms of the squared log of odds ratio, and further allowed for the effect size to have different values for different genuine signals, assuming a continuous (gamma) distribution. We assumed the proportion of null signals is known and considered three degrees of the knowledge regarding the effect size distribution:

1. Complete knowledge, i.e., precise fraction of signals with the odds ratio greater than any given magnitude. Note that the knowledge of the distribution does not imply knowledge of the effect size that gave rise to any given P-value. Only summary information given by a probability distribution of various odds ratios is assumed. We refer to this as the exact or the integration method.
2. Knowledge about the abundance of three classes of odds ratios, low, average and high. We refer to this as the discretization or the tabulated values method. In this paper, we approximate the effect size distribution by three bins, capturing the bulk of the effect size distribution and the fourth “null” bin containing close-to-zero effect sizes, as illustrated in Fig 1. Posterior probabilities in these simulations were evaluated by Eq (3), assuming that the null bin includes odds ratios of 1 to 1.001. We also evaluated these probabilities by Eq (1) that assumes the sharp null hypothesis. Results obtained by these two methods were identical (up to the reported precision). Note that the simulations also modeled the null hypothesis as sharp, i.e., the expected proportion Pr(*H*_0_) of all signals in a simulation experiment had exactly zero effect size.
3. Knowledge about only the mean of the distribution. We refer to this as the single-value or the distribution mean method.

In our experiments we focused on estimating POFIG probabilities for the smallest P-values. Selection of the smallest P-values may cause bias in estimation. An extreme case of selection is when the single smallest P-value is selected from a multiple testing experiment, which we call the minP. Additionally, we may envision a process whereby the entire experiment is discarded by a researcher unless the minP is smaller than the multiple testing adjusted significance threshold. Thus, we considered two types of P-value selection (1) selecting the smallest P-values of every experiment and (2) selecting the smallest P-values of only those experiments where statistically significant findings were observed. First, we considered just the single smallest P-value, the minP, to determine whether our method can correctly recover the probability that the minP represents a spurious signal in both of these selection scenarios. Next, we extended the experiments by considering a larger set of the smallest P-values. In these experiments, we calculated the proportion of spurious signals among selected smallest P-values and evaluated how well our method can recover that proportion. We used either the Bonferroni at the level of 0.05/*K* or Benjamini and Hochberg’s FDR criterion [12] at the level of 10% as the rule for discarding sets of the smallest P-values.

#### 2.1.1 Posterior probability of *H*_0_ for a signal with the smallest P-value with or without a multiple testing correction

Table 1 summarizes the posterior probabilities that the smallest P-value is a spurious signal in a study with *K* tests. As expected, the posterior probabilities are unaffected by the “winner’s curse” phenomenon: the true (first number in each cell) and the estimated probabilities are nearly identical for the “exact” method (the second entry in each cell) which assumes precise knowledge of the effect size distribution (the number of simulations was 50,000 for Table 1 and 10,000 for other tables). The three-value approximation works well but becomes conservatively biased with 1 × 10^6^ tests; the bias increases for Bonferroni-selected scans (the “0.05/*K*” columns) compared to experiments without selection and for experiments with the relatively higher proportion of non-zero signals. The single value approximation (last number in each cell) is surprisingly good for small to moderate number of tests. At 1 × 10^6^ tests, the bias for the smallest P-value becomes excessive (Table 1), but the bias is greatly reduced as P-values with higher ranks are considered (Table 2). Results in Table 1 suggests that the Bonferroni adjustment for multiplicity (*α* = 0.05/*K* columns) fails to substantially decrease the probability that the smallest P-value is a spurious finding: although we observe a decrease in the values of posterior probability, the reduction is not as drastic as may have been anticipated, considering the stringency of the Bonferroni threshold, 0.05/*K*.

**Table 1:** Probability that the minimum P-value is a false finding. Table entries W/X/Y/Z give the true probability (W), followed by the estimated probabilities using the integration method (X); the discretization method (Y); the distribution mean method (Z). Sample sizes (*n*_1_, *n*_2_: number of cases, controls in allele counts): *n*_1_ + *n*_2_ = 800 for *K* = 10 to 10,000; *n*_1_ + *n*_2_ = 4000 for *K* = 10^6^.

Number of tests	Pr(*H*_0_) = 0.5		Pr(*H*_0_) = 0.9	
	No correction	*α* = 0.05/*K*	No correction	*α* = 0.05/*K*
*K* = 10	0.40/0.40/0.40/0.40	0.26/0.26/0.26/0.27	0.86/0.86/0.86/0.86	0.76/0.76/0.76/0.77
*K* = 100	0.28/0.28/0.28/0.29	0.18/0.18/0.18/0.20	0.79/0.79/0.79/0.80	0.67/0.67/0.67/0.70
*K* = 1000	0.19/0.19/0.19/0.21	0.12/0.12/0.13/0.16	0.70/0.70/0.71/0.73	0.56/0.56/0.58/0.62
*K* = 10,000	0.12/0.12/0.13/0.15	0.08/0.08/0.09/0.12	0.59/0.59/0.61/0.65	0.45/0.45/0.48/0.55
	Pr(*H*_0_) = 1-1000/10^6^		Pr(*H*_0_) = 1-150/10^6^	
	No correction	*α* = 0.05/*K*	No correction	*α* = 0.05/*K*
*K* = 10^6^	0.36/0.36/0.47/0.74	0.13/0.13/0.23/0.58	0.84/0.84/0.89/0.97	0.51/0.51/0.68/0.91

**Table 2:** The average of posterior probabilities among the 100 smallest P-values, i.e., the false discovery rate (FDR) among the 100 smallest P-values. Table entries W/X/Y/Z give the true averaged probability (W), followed by the estimated averaged probabilities using the integration method (X); the discretization method (Y); the distribution mean method (Z). Sample sizes (*n*_1_, *n*_2_: number of cases, controls in allele counts): *n*_1_ + *n*_2_ = 800 for *K* = 10 to 10,000; *n*_1_ + *n*_2_ = 14000 for *K* = 10^6^.

Number of tests	Pr(*H*_0_) = 0.5	Pr(*H*_0_) = 0.9
*K* = 100	0.50/0.50/0.50/0.50	0.90/0.90/0.90/0.90
*K* = 500	0.42/0.42/0.42/0.42	0.87/0.87/0.87/0.87
*K* = 1000	0.38/0.38/0.38/0.38	0.85/0.85/0.85/0.85
*K* = 10,000	0.26/0.26/0.26/0.27	0.78/0.78/0.78/0.79
	Pr(*H*_0_) = 1-1000/10^6^	Pr(*H*_0_) = 1-150/10^6^
*K* = 10^6^	0.17/0.17/0.16/0.23	0.82/0.82/0.81/0.84

#### 2.1.2 Proportion of false signals among the 5 smallest P-values with and without an application of the FDR criterion

Similar conclusions can be drawn from Table 3 where Benjamini and Hochberg’s FDR criterion [12] (B&H’s FDR) was applied at the level of 10%. In experiments with the application of B&H’s FDR, the five smallest P-values were discarded unless all five satisfied B&H’s FDR criterion. Proportions of spurious signals are very similar for the results with and without the application of B&H’s FDR criterion.

**Table 3:** The average of posterior probabilities among the 5 smallest P-values, i.e., the false discovery rate (FDR) among the 5 smallest P-values. Table entries W/X/Y/Z give the true probability (W), followed by the estimated probabilities using the integration method (X); the discretization method (Y); the distribution mean method (Z). Sample sizes (*n*_1_, *n*_2_: number of cases, controls in allele counts): *n*_1_ + *n*_2_ = 800.

	Pr(*H*_0_) = 0.5		Pr(*H*_0_) = 0.9	
Number of tests	No correction	B&H’s FDR at 10%	No correction	B&H’s FDR at 10%
*K* = 10	0.47/0.47/0.47/0.47	0.36/0.36/0.36/0.36	0.89/0.89/0.89/0.89	0.84/0.83/0.83/0.83
*K* = 100	0.35/0.35/0.35/0.35	0.26/0.26/0.26/0.27	0.83/0.83/0.83/0.84	0.75/0.76/0.76/0.77
*K* = 1000	0.23/0.23/0.24/0.25	0.18/0.18/0.19/0.20	0.75/0.76/0.76/0.77	0.66/0.66/0.67/0.70
*K* = 10,000	0.15/0.15/0.16/0.18	0.13/0.12/0.13/0.15	0.66/0.65/0.67/0.69	0.56/0.56/0.58/0.63

#### 2.1.3 Proportion of false signals among the 100 smallest P-values

Table 2 summarizes the proportion of false signals among the set of 100 smallest P-values. That proportion, or the false discovery rate (FDR), is simply the average of posterior probabilities among the 100 smallest P-values. In this case, even the single-value approximation is reasonably close to the true value: as the P-value rank moves away from minP, the bias decreases.

Interestingly, the three tables of simulation results demonstrate the phenomenon that as the number of tests becomes larger, the proportion of spurious signals among a given number of top hits decreases. This can be inferred from inspecting results of experiments with the numbers of tests *K* increasing from 10 to 10,000, where the sample size and the prior Pr(*H*_0_) were held the same for different values of *K*.

#### 2.1.4 POFIGs for the smallest P-value from a scan, its replication P-value, and for the combined scan and replication P-value

**Fig 2:**
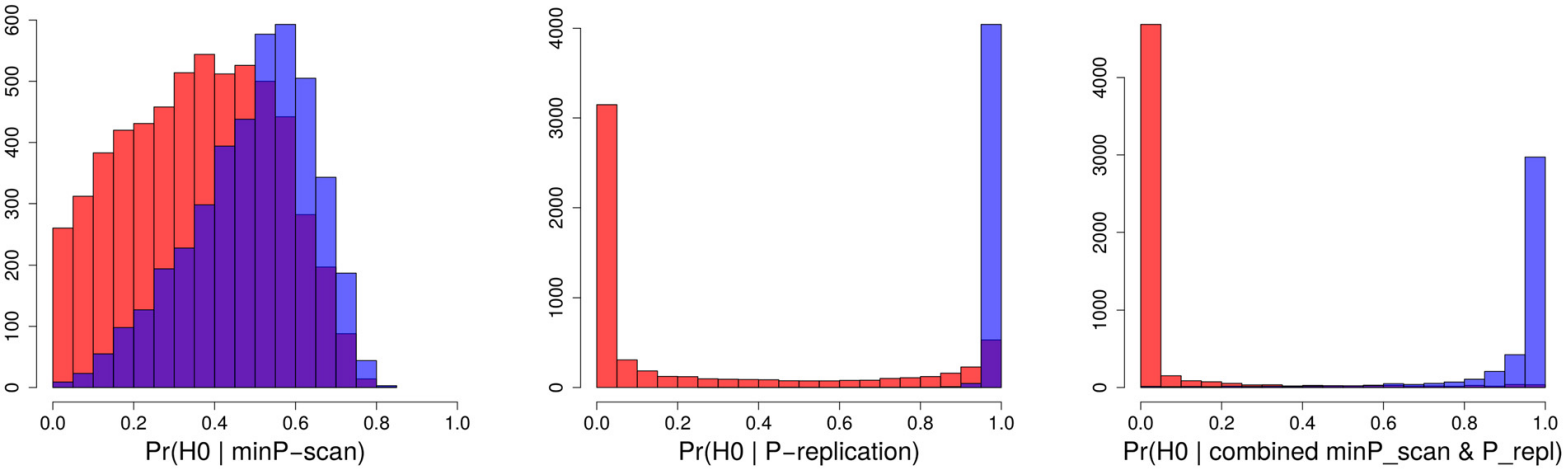
Distribution of estimated posterior probabilities that a P-value is a false finding. Color refers to whether the actual signal is genuine (red) or false (blue). Purple color denotes the overlap of genuine and false signals. **First graph**: Estimated probability that the smallest P-value in a scan (minP) is a false finding. **Second graph**: Estimated probability that the signal from the scan with the smallest P-value is a false finding when evaluated in a replication study. **Third graph**: Estimated probability that the finding is false based on the combined P-value (the smallest scan P-value combined with the replication P-value).

In Fig 2, the first graph is a histogram of estimated posterior probabilities associated with the smallest P-value for a simulated discovery scan with 10,000 tests (simulation details are described in the Methods section “Scan, replication and combined probabilities”). Smaller probabilities that the finding is false are associated with the over-presence of genuine signals, indicated by red color, while large probabilities are associated with false signals, indicated by blue color. The mean value of the first histogram does estimate the true proportion of false signals in an unbiased way. However, there is a large area of overlap (purple color), that is, there is no good separation of “red” and “blue” signals. Once all of these signals are evaluated in a four times larger replication sample (middle histogram), we observe an appropriately U-shaped distribution. Now most low and high probabilities correctly capture the genuine and the false signals, respectively, although there is a sizable purple bar at the right indicating mis-classification of some genuine signals as false. We observe that a sufficiently large sample size assures a good correspondence between the probability and the actual status (genuine vs. false) of a signal. The U-shape becomes more prominent in the last histogram that is based on probabilities for the combined scan and replication P-values, due to an increased sample size. These figures clearly demonstrate utility of POFIG for discrimination of genuine and spurious signals. A value of Pr(*H*_0_|P-value) that is close to one reflects high likelihood that the finding is spurious. In contrast to that, large association P-value does not give support toward the hypothesis of no association.

### 2.2 POFIG application to Crohn’s disease P-values

For the analysis of real data, we used SNP association P-values for Crohn’s disease from Barrett et al.[13] We considered newly identified loci from Table 2 of their work, selecting those SNPs for which genes of interest were listed. Utilizing their scan and replication P-values, we applied the POFIG method to provide scan and replication posterior probabilities of no association for selected SNPs. We used the prior for the *H*_0_ equal to 1-142/12877, where 142 is the number of susceptibility loci estimated by Park et al.[7] and 12877 is the total number of loci, estimated using their definition of a locus based on the extent of linkage disequilibrium. We assumed the effect size distribution to be a gamma with the shape parameter 1 and the scale parameter was chosen to give the distribution mean computed from Supplementary Table 3 of Park et al. We also conducted a sensitivity analysis, utilizing different priors and effect size distributions including tabulated effect sizes given in Park et al.[7].

It is common to combine P-values or association statistics between studies, for example, as presented in Barrett et al.’s Table 2. It should be pointed out that although this type of meta-analysis, based on summary statistics can be nearly as efficient as analysis based on pooling raw data [14], possible non-independence of samples and effect size heterogeneity between studies require careful consideration during meta-analysis of GWAS [15].

The scan and replication probabilities can also be combined. These combined probabilities can be obtained by using the posterior probability from the scan as the prior Pr(*H*_0_) for the replication. This is mathematically equivalent to replacement of $f_{\bar{\gamma }}(p)$ in Eq (1) by the product $f_{\bar{\gamma }}(p_{1})\times f_{\bar{\gamma }}(p_{2})$ where *p*_1_ and *p*_2_ are the scan and the replication P-values, while using the initial prior probability, Pr(*H*_0_), for the scan. Using this approach, combined posteriors probabilities, labeled as “Combined Pr(*H*_0_|P-value)”, were obtained by combining the scan and the replication posterior probabilities that the finding is spurious for the newly discovered SNP associations. The results based on the combined probabilities provide substantially higher chances that the newly identified loci are genuine findings. Barrett et al. reported combined P-values for the scan and the replication statistics. Their values were based on a combination of two-sided statistics. Therefore, in our analyses, both the scan- and the replication posterior probabilities were obtained using two-sided P-values. This approach does not take into account direction of the association (i.e., effect sign). In Materials and Methods we describe a simple way that takes into consideration the correspondence of effect directions between studies (section “Scan, replication and combined probabilities”).

**Fig 3:**
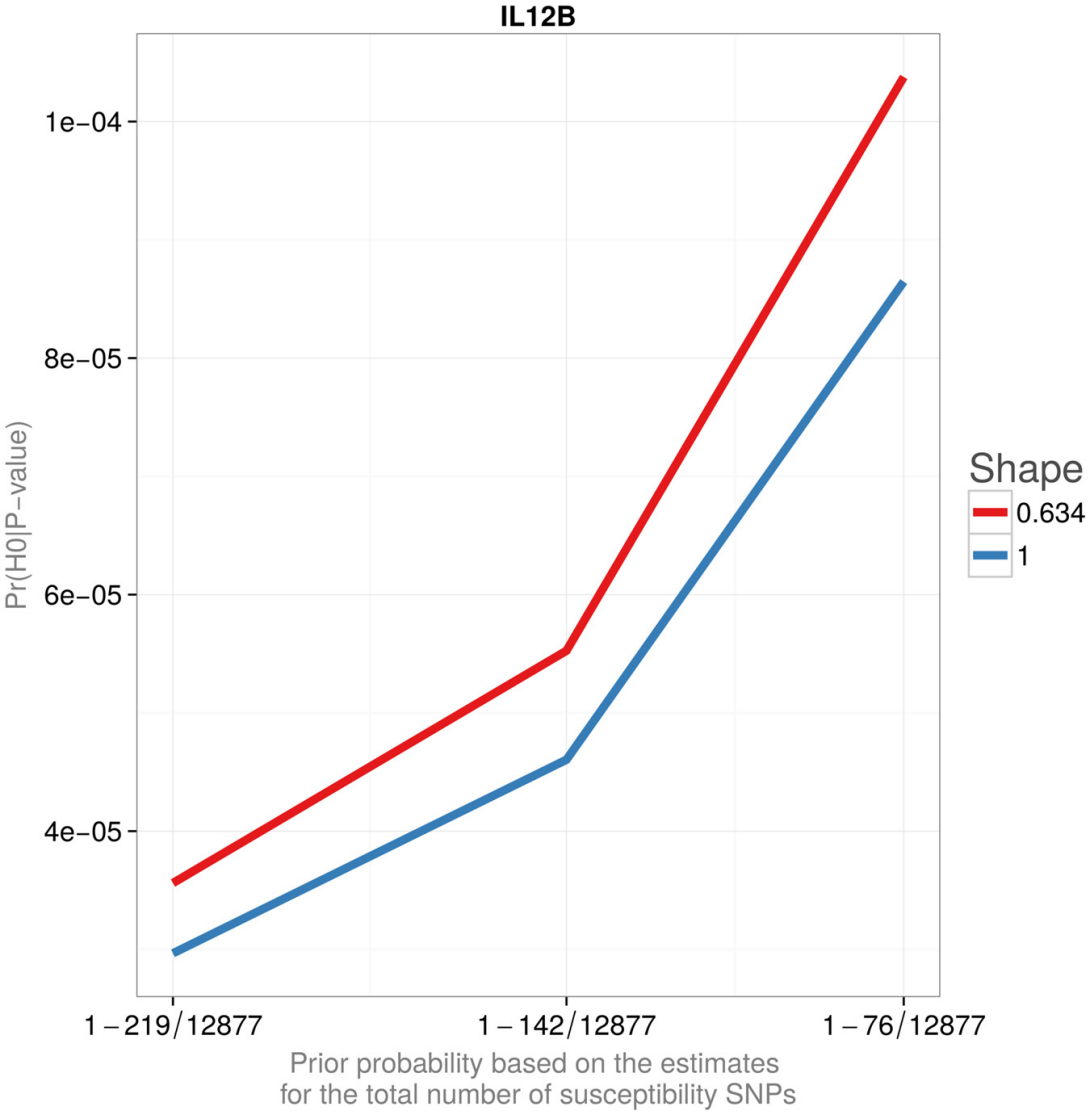
Minus log of posterior probabilities that the finding is spurious. Minus 1-POFIG for novel SNP associations with Crohn’s disease in Barrett et al. reported on the logarithmic scale.

**Fig 4:**
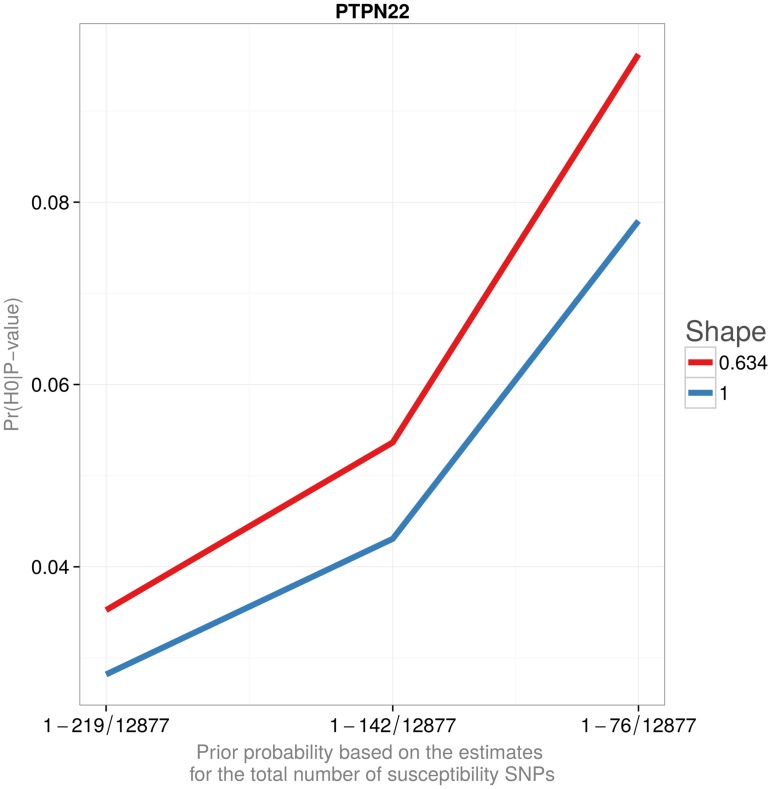
Probability that a finding is false (1-POFIG). The probability that the most significant newly identified loci reported in Barrett et al. is false. The prior probabilities are based on the estimates for the total number of susceptibility SNPs reported in Park et al. The shape parameter 0.634 was chosen to yield the median value of the noncentrality distribution to be twice as small as that of the exponential distribution (Shape = 1) for the same value of the scale.

**Fig 5:**
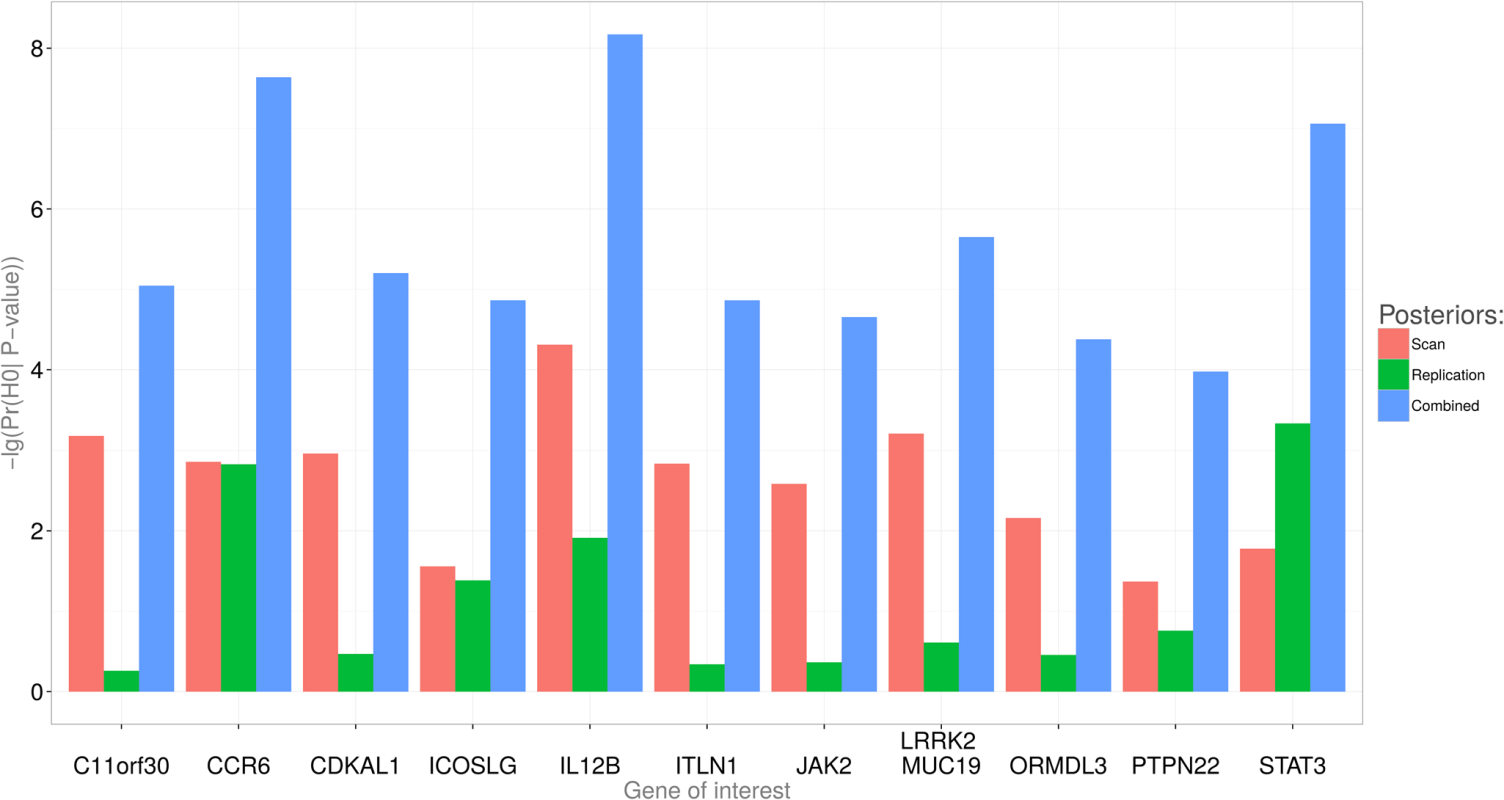
Probability that a finding is false (1-POFIG). The probability that the least significant newly identified loci reported in Barrett et al. is false. The prior probabilities are based on the estimates for the total number of susceptibility SNPs reported in Park et al. The shape parameter 0.634 was chosen to yield the median value of the noncentrality distribution to be twice as small as that of the exponential distribution (Shape = 1) for the same value of the scale.

Fig 3 and Table 4 provide combined posterior probabilities for the newly identified loci reported in Barrett et al.[13]. Barrett et al.’s scan, replication, and combined P-values are given in the table for comparison. Figs 4 and 5 and Table 5 illustrate the effect of the prior probability and the assumed effect size distribution on the posterior estimates of Pr(*H*_0_|P-value). Neither of the choices considered in the Table and Figures substantially affected the posterior probability that the association is false. In particular, posterior probabilities using the tabulated distribution are similar to the ones obtained assuming a continuous, L-shaped gamma distribution.

**Table 4:** P-values reported for the newly identified loci for Crohn’s disease in Barrett et al. and the corresponding posterior probabilities of the null hypothesis. Rows highlighted in bold correspond to the maximum and minimum Scan P-values.

	P-value			Pr(*H*_0_|P-value)			
SNP	Scan	Replic.	Combined	Scan	Replic.	Combined	Gene
**rs2476601**	**1.8** × **10** ^**-5**^	**1.0** × **10** ^**-4**^	**1.5** × **10** ^**-8**^	**0.043**	**0.174**	**1.1** × **10** ^**-4**^	PTPN22
rs2274910	3.5 × 10^−7^	4.8 × 10^−4^	1.5 × 10^−9^	0.001	0.457	1.4 × 10^−5^	ITLN1
**rs10045431**	**8.8** × **10** ^**-9**^	**3.7** × **10** ^**-6**^	**3.9** × **10** ^**-13**^	**4.9** × **10** ^**-5**^	**0.012**	**6.7** × **10** ^**-9**^	IL12B
rs6908425	2.5 × 10^−7^	2.8 × 10^−4^	9.0 × 10^−10^	0.001	0.340	6.3 × 10^−6^	CDKAL1
rs2301436	3.3 × 10^−7^	3.3 × 10^−7^	1.0 × 10^−12^	0.001	0.001	2.3 × 10^−8^	CCR6
rs10758669	6.8 × 10^−7^	4.3 × 10^−4^	3.5 × 10^−9^	0.003	0.432	2.2 × 10^−5^	JAK2
rs7927894	1.4 × 10^−7^	7.3 × 10^−4^	1.3 × 10^−9^	0.001	0.551	9.0 × 10^−6^	C11orf30
rs11175593	1.3 × 10^−7^	1.6 × 10^−4^	3.1 × 10^−10^	0.001	0.245	2.2 × 10^−6^	LRRK2-MUC19
rs2872507	2.1 × 10^−6^	2.9 × 10^−4^	5.0 × 10^−9^	0.007	0.350	4.2 × 10^−5^	ORMDL3
rs744166	5.9 × 10^−6^	9.1 × 10^−8^	6.8 × 10^−12^	0.017	4.6 × 10^−4^	8.7 × 10^−8^	STAT3
rs762421	1.1 × 10^−5^	1.6 × 10^−5^	1.4 × 10^−9^	0.028	0.041	1.4 × 10^−5^	ICOSLG
The proportion of false findings (FDR)				0.009	0.237	1.9 × 10^−5^	

**Table 5:** Posterior Pr(*H*_0_|P-value) for two newly identified loci based on the results reported in Barrett et al. [13]. The three prior probabilities are based on the three estimates for the total number of susceptibility SNPs taken from Park et al’s. supplementary Table 5. [7] The “Tabulated” effect size distribution is based on Park et al’s supplementary Table 3; “scale” in Gamma(1 or 0.634, scale) is chosen to have the distribution mean equal to that of the tabulated distribution.

SNP	Gene	Reported P-value	Prior	Effect size distribution	Posterior
rs10045431	IL12B	8.80 × 10^−9^	1-76/12877	Tabulated	1.9 × 10^−4^
				Gamma(0.634, 27.21)	1.0 × 10^−4^
				Gamma(1, 17.25)	8.6 × 10^−5^
			1-142/12877	Tabulated	9.9 × 10^−5^
				Gamma(0.634, 27.21)	5.5 × 10^−5^
				Gamma(1, 17.25)	4.6 × 10^−5^
			1-219/12877	Tabulated	6.4 × 10^−5^
				Gamma(0.634, 27.21)	3.6 × 10^−5^
				Gamma(1, 17.25)	3.0 × 10^−5^
rs2476601	PTPN22	1.81 × 10^−5^	1-76/12877	Tabulated	0.082
				Gamma(0.634, 27.21)	0.096
				Gamma(1, 17.25)	0.078
			1-142/12877	Tabulated	0.045
				Gamma(0.634, 27.21)	0.054
				Gamma(1, 17.25)	0.043
			1-219/12877	Tabulated	0.030
				Gamma(0.634, 27.21)	0.035
				Gamma(1, 17.25)	0.028

**Fig 6:**
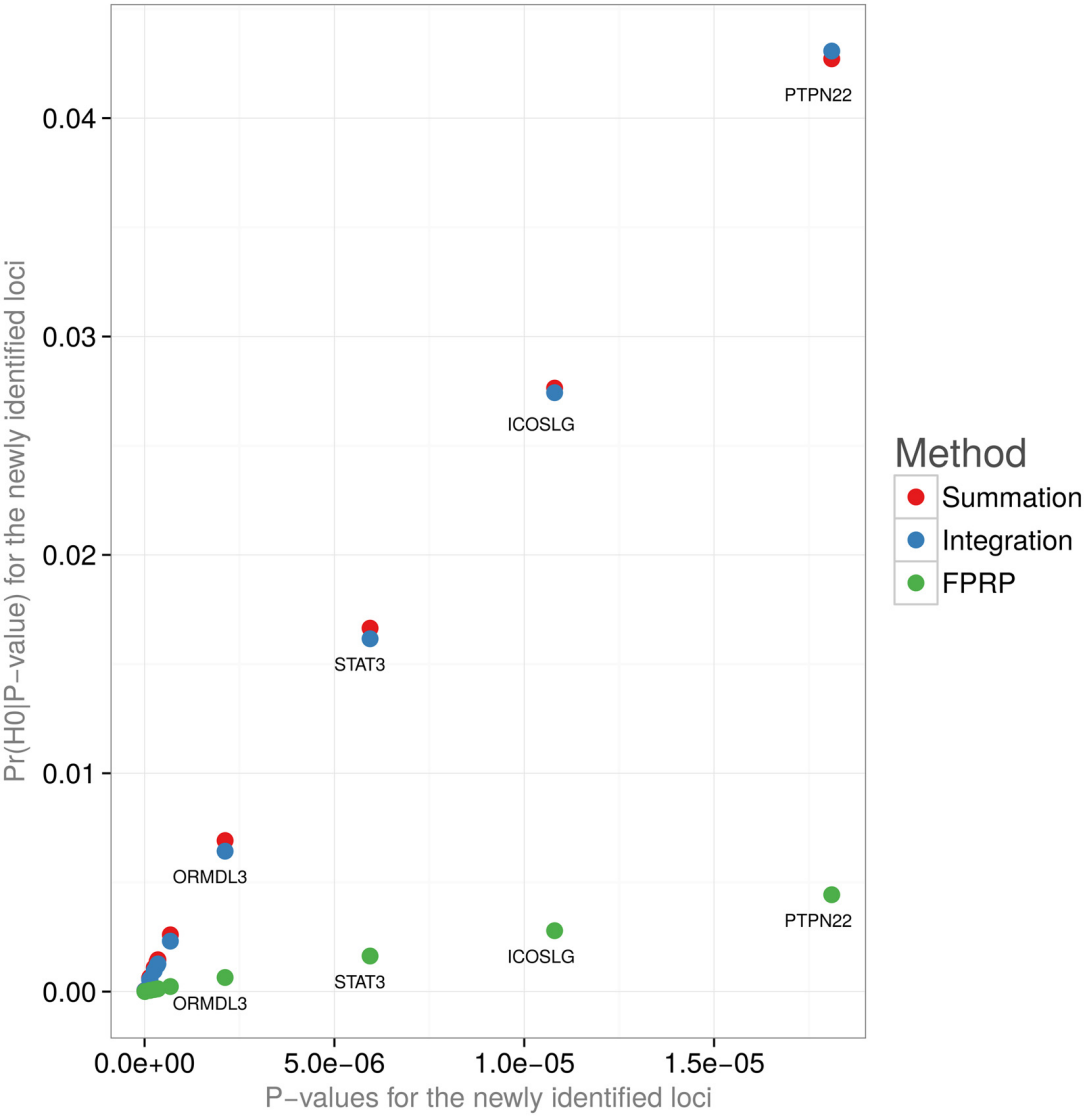
Posterior probabilities of the null hypothesis by POFIG and FPRP methods. The posterior probabilities are calculated for the newly identified Crohn’s disease associations from Barrett et al.’s study [13]. Unmarked genes in the lower left corner: IL12B, LRRK2-MUC19, C11orf30, CDKAL1, CCR6, ITLN1—for red and blue; IL12B, LRRK2-MUC19, C11orf30, CDKAL1, CCR6, ITLN1, JAK2—for green (FPRP).

Fig 6 illustrates the comparison of the POFIG posterior probabilities of no true associations to the FPRP values. The FPRP estimates tend to be smaller, as they refer to the whole tail of P-values as small as the one observed. The posterior probabilities of *H*_0_ are monotone in the ranking of P-values as the distribution of the noncentralities for this analysis was specified directly (Eq 14), based on the results reported in Park et al.[7]

## 3 Discussion

Modern molecular technologies continue to reveal information about genetic architecture of human diseases. It is now possible to utilize available knowledge to make informed decisions regarding parameters needed for the calculation of probability that a P-value represents a genuine association, namely the overall proportion of susceptibility loci and the distribution of their effect sizes. The “False Positive Report Probability” (FPRP) approach [3] utilizing these parameters was proposed previously, however the FPRP was not designed as a method for assessing the probability that a finding (i.e., a “report”) in question is false. Here we introduce a similarly inspired approach, the method for assessing the “PrObability that a FInding is Genuine” (POFIG). Our method eliminates the shortcomings of FPRP, but shares its computational simplicity.

As in the FPRP approach, one can simply assume a “typical” effect size (taken to be the mean of the underlying true distribution). This results in a method that is just as simple computationally as the FPRP. Its bias in the estimation of the posterior probability increases with the number of tests, but decreases with the P-value magnitude or rank. That is, the bias is the largest for the most significant finding in a study. When the mean value is used, estimate of the posterior probability that the finding is spurious appears to be conservative (i.e., it is larger than the true value) and is tolerably small for experiments with as many as 10,000 tests. Truly massive multiple testing experiments such as whole genome sequencing and genome-wide association studies may require a more precise specification of the effect size distribution. Ideally, the whole probability distribution needs to be specified and the population genetics theory suggests that a gamma distribution can be successfully utilized to model the expected L-shaped distribution of effect sizes [16]. A far more convenient way is to assume several tabulated effect size values with the relative abundance of each. Methods and data from which these values can be readily extracted are starting to emerge [7]. We find that this computationally simple approach can greatly reduce the bias and usage of only three bins with low, moderate and large effect size values already provides sufficient precision.

False signals can be handled in two different ways within our approach. Traditionally, false signals are assumed to have zero effect size; this requires specification of the prior probability of the null hypothesis, Pr(*H*_0_). Alternatively, an extra “null bin” is introduced with its respective proportion for signals which effect sizes are within a narrow interval close to zero. The second specification acknowledges an understanding that there are many signals whose effect sizes are tiny but non-zero, avoiding an unrealistic assumption that there is a sizable fraction of signals with exactly zero effect size.

There are various desirable properties of POFIG. When the same genetic variant (hypothesis) is tested in several studies, one can assess the combined posterior probability of false association by sequentially using the 1-POFIG probability from one study as the prior Pr(*H*_0_) for the next. This can be entered in any order and is equivalent to using the initial prior probability, Pr(*H*_0_), while replacing *f*_*γ*_(*p*) in Eq (1) by the product *f*_*γ*_(*p*_1_) × *f*_*γ*_(*p*_2_) × …, where *p*_*i*_ is the *i*-th study P-value. Alternatively, one can combine study-specific P-values first and convert the result to the posterior probability of a hypothesis. This approach gives a simple way to account for correspondence in the effect direction between studies (Eqs 20, 21). We may contrast this approach with the practice of combining scan and replication statistics or P-values. Barrett et al. reported scan, replication and combined P-values in their Table 2. Their combined values are obtained by pooling Z-statistics, and very similar values can be obtained by combining their scan and replication P-values by the inverse normal method [17]. These combined P-values are biased under the hypothesis of no association by the fact that the scan P-values were selected among the set of the smallest P-values. To preserve the type-I error rate in a conservative way, these combined P-values would need to be adjusted by the number of tests in the scan. Ideally, classical P-value combination procedures would need to take into account the number of tests as well as ranks of P-values in both the scan and the replication studies [18].

Following our approach, we combined the scan and the replication posterior probabilities for novel SNP associations with Crohn’s disease [13] using our approach (Fig 3). Not only the two probabilities can be combined in a straightforward way, but they can also be averaged across selected SNPs. This average gives the estimated proportion of spurious signals among the SNPs. In the analysis of Barrett’s data, we computed probabilities that a finding is spurious (i.e., false). The average of these probabilities gives the estimated proportion of false signals, or the false discovery rate estimate (FDR), as reported in the last row of Table 4.

Selection issues aside, the scan and combined P-values in Table 4 should be viewed with caution, because the smallest GWAS P-values may themselves be imprecise for various reasons, including residual confounding, genotyping errors, and violations of statistical assumptions in computing P-values from very large values of association statistics. We also assume that the bulk of P-value distribution is appropriately flattened by methods that adjust for systematic biases caused by population stratification or other sources of confounding [19–21]. Our posterior probabilities are extracted from P-values, thus they are similarly affected by these issues.

Our approach compares favorably to classical estimation procedures where effect size estimates for top hits of a study tend to be over-estimated and the corresponding P-values tend to be too small. This bias, known as the winner’s curse phenomenon, has been a formidable challenge. Bowden and Dudbridge concluded that in the classical (frequentist) statistical framework, no unbiased correction is possible without replication data [10]. Combination of selected smallest P-values results in complicated distributions [22–24] and methods for combining results from studies, some of which involve selection of the best results, are lacking. The main appeal of our method is that it is unaffected by selection of either smallest or statistically significant P-values, as we demonstrated by simulation experiments. Estimated posterior probabilities and their averages among selected P-values (FDR estimates) are unbiased. These estimates of posterior probabilities and especially of their averages among the smallest P-values were shown to be robust in the presence of high linkage disequilibrium [25]. Admittedly, high precision in estimation of true probabilities comes at a price of correct specification of the effect size distribution and the overall proportion of susceptibility variants. This underlines importance of emerging statistical research on estimation of these parameters from large scale genetic data.

Table 1 shows that in experiments with and without a multiplicity correction, the smallest P-value is increasingly more likely to represent a genuine signal as the number of tests increases from 10 to 10,000. In experiments with the multiplicity correction, only those experiments were retained for analysis where one or more results were significant. Although the multiplicity adjustment itself does not change the order of P-values, probability for the signal with the smallest P-value to be genuine changes when computed among those retained experiments with significant findings. There is another interpretation of these results. In the columns with the multiplicity correction, simulations can be thought of as modeling a process that leads to the publication bias phenomenon: experiments without significant findings are put in a file drawer, while those with significances are sent to a journal. The fact that the calculated posterior probability correctly estimates the true proportion of spurious results indicates that our method is unaffected by the publication bias phenomenon. The calculation of posterior probabilities is not informed in any way by the significance threshold used or by how many tests were performed.

It is understood within the research community that P-values, even after a multiplicity adjustment still do not reflect chances that the finding is spurious. Nevertheless, multiplicity adjustments are thought to be highly efficient in filtering out spurious results, similarly to how the distillation process turns the wash into vodka. It is evident from Table 1 after comparing probabilities for columns with and without the correction that the multiplicity adjustment even as strict as the Bonferroni does not necessarily serve as a good filter of spurious signals. Application of Benjamini and Hochberg’s FDR criterion (Table 3) changes the true proportion of spurious signals only slightly. When half of the signals are genuine, this proportion drops from 15% to 12%, but in these simulations, only about 1 in every 18 experiments had all five smallest P-values significant by this criterion. These simulations were deliberately designed to be under-powered to detect any single genuine signal. In studies with low power, genuine signals do not come up at the top with high certainty. In these situations, multiple-testing approaches should supposedly shield us from accepting too many spurious signals as genuine. As we see, this is not the case. Moreover, there appears to be increased enrichment of top hits by genuine signals as the number of tests becomes larger.

In summary, P-values, despite their deficiencies, do contain information that can be used for judging validity of findings and we propose POFIG for converting P-values to valid probabilities that a finding is genuine. As we demonstrate, this simple method avoids numerous complications that arise in making decisions based on P-values. POFIG requires an informed input from researchers regarding a likely proportion of genuine signals in their multiple testing experiments and some characterization of the effect size distribution. The later can be replaced by a good guess for the mean of that distribution unless the P-values are extremely small, in which case validity of P-values themselves is likely in doubt.

## Software

Microsoft Excel and R programs to convert P-values to posterior probabilities that a finding is genuine are available at https://sites.google.com/site/pofigscript/ or by a request to D.V.Z. The programs assume that P-values result from one degree of freedom chi-square tests, but can be easily modified to accommodate other tests statistics. We welcome requests for help with modifications.

## 4 Materials and Methods

Suppose our test statistic is *Z*, with the cumulative distribution function (CDF) under the null hypothesis denoted by *G*_0_(·). Under the alternative hypothesis, *H*_*A*_, we denote its CDF by *G*_*γ*_(·). The parameter *γ* captures deviation from the null hypothesis. P-value is obtained in the usual manner, as *P* = 1 − *G*_0_(*Z*). By rewriting this and applying the inverse of the CDF, $G_{0}^{-1}(\cdot )$, we recover the statistic *Z*:

$$\begin{array}{ccc} 1-P & = & G_{0}\left(Z\right)\\ G_{0}^{-1}\left(1-P\right) & = & Z \end{array}$$ (7)

Under *H*_*A*_, the P-value has the CDF 1 − *G*_*γ*_(*Z*). We can apply 1 − *G*_*γ*_(·) to the both sides of Eq (7) to write this distribution in terms of the usual P-value computed under *H*_0_. Thus, under *H*_*A*_, the P-value CDF, which we denote by *F*_*γ*_(*p*) is

$$\begin{array}{c} F_{\gamma }\left(p|\gamma \right)=1-G_{\gamma }[G_{0}^{-1}\left(1-p\right)] \end{array}$$ (8)

By differentiating this CDF, we obtain the corresponding probability density function, PDF:

$$\begin{array}{ccc} f_{\gamma }\left(p|\gamma \right) & = & \frac{g_{\gamma }(G_{0}^{-1}\left(1-p\right))}{g_{0}(G_{0}^{-1}\left(1-p\right))} \end{array}$$ (9)

Here, *g*_0_(·) and *g*_*γ*_(·) are the PDFs that correspond to the CDFs *G*_0_(·) and *G*_*γ*_(·).

P-value distribution as defined in this section arises from a broad range of statistical tests. For a normally distributed test statistic, *γ* is the normal mean, shifted away from zero. For chi-squared *F*, and *t* statistics, *γ* is the noncentrality parameter of the corresponding distribution. In all these cases, zero value of *γ* corresponds to *H*_0_. Thus, the P-value distribution is quite general with regard to the underlying statistical test. Its applicability is not limited to models with a single predictor; e.g., in regression models with adjustment for covariates, distribution of P-value for a predictor of interest can be modeled in the same way. The proposed approach applies to continuous test statistics with a distribution where the degree of deviation from *H*_0_ is captured by a value of a single parameter (*γ*). Because this approach requires an explicit specification of the distribution, it cannot be readily applied to P-values that result from permutation-based tests and other tests that are based on resampling. It is at present unclear whether our approach would provide sufficient precision when applied simply as a convenient statistical device to approximate and model an unknown (e.g., resampling) distribution. We note that a beta distribution has been used successfully to mimic P-value distributions in several studies concerned with problems related to those considered here [26–29]. The choice of a beta distribution in these studies was due to flexibility of its shape rather than to any known relation to the actual distribution of P-values. Good performance of approximations based on a beta distribution is encouraging, given that the P-values of common test statistics do not in fact follow that distribution. Thus, the issue of applicability of our approach to P-values that do not necessarily follow an assumed parametric distribution is worthy of future investigation.

To make the exposition more focused, we primarily discuss the binary outcome, a chi-square statistic, and the effect size defined via odds ratios or directly as a value of the noncentrality, but there is no restriction for the outcome to be binary. Lee and Wray provide useful expressions that present the noncentrality parameter in terms of the proportion of variability in the outcome due to a genetic predictor [30]. These expressions cover a variety of designs with different sampling schemes and both continuous and binary outcomes. Yang et al. give formulas to relate noncentralities of population-based quantitative trait and case-control designs [31].

### 4.1 False Positive Report Probability

If the rejection of the null hypothesis is based on some nominal *α*-level, such as 5%, the probability of obtaining P-value, *P*, that is at least as small as *α* is equal to *α*, given that the null hypothesis is true: Pr(*P* ≤ *α*|*H*_0_) = *α*. The value *α* defines the type-I error rate. Under the alternative hypothesis, denoted by *H*_*A*_, the corresponding probability is the power: Pr(*P* ≤ *α*|*H*_*A*_) = 1 − *β*, where *β* is the type-II error rate: the probability of failing to reject the null hypothesis correctly. By the Bayes rule,

$$\begin{array}{ccc} \mathrm{Pr}(H_{0}|P\leq \alpha ) & = & \frac{\mathrm{Pr}\left(P\leq \alpha |H_{0}\right)\mathrm{Pr}\left(H_{0}\right)}{\mathrm{Pr}\left(P\leq \alpha |H_{0}\right)\mathrm{Pr}\left(H_{0}\right)+\mathrm{Pr}\left(P\leq \alpha |H_{A}\right)\mathrm{Pr}\left(H_{A}\right)}\\ & = & {\left[1+\frac{1-\mathrm{Pr}(H_{0})}{\mathrm{Pr}(H_{0})}\times \frac{\mathrm{Pr}(P\leq \alpha |H_{A})}{\mathrm{Pr}(P\leq \alpha |H_{0})}\right]}^{-1} \end{array}$$ (10)

This approach was used by Morton to define posterior probabilities in the context of genetic linkage analysis [32]. It also forms the basis of the false positive report probability (FPRP)[3]. Although Wacholder et al. assumed a single (typical) effect size, the probability Pr(*P* ≤ *α*|*H*_*A*_) depends on the distribution of all possible effect sizes and has to be represented in its marginal form, i.e., averaged over all possible values of the effect size for genuine signals

$$\begin{array}{c} \mathrm{Pr}\left(P\leq \alpha |H_{A}\right)=\int F_{\gamma }\left(\alpha |\gamma \right)\Gamma \left(\gamma \right)d\gamma \end{array}$$ (11)

assuming that *γ* follows the distribution Γ(*γ*). By using the notation $F_{\bar{\gamma }}(\alpha )=\mathrm{Pr}(P\leq \alpha |H_{A})$ and assuming, following Wacholder et al., uniformity of P-values under *H*_0_, so that *F*_0_(*α*) = Pr(*P* ≤ *α*|*H*_0_) = *α*, the FPRP is

$$\begin{array}{c} \text{FPRP}={\left[1+\frac{1-\mathrm{Pr}(H_{0})}{\mathrm{Pr}(H_{0})}\times \frac{F_{\bar{\gamma }}\left(\alpha \right)}{\alpha }\right]}^{-1} \end{array}$$ (12)

Eq (12) gives the proportion of null signals among P-values that are smaller or equal to a fixed threshold *α*. Wacholder et al. advocated plugging in an observed P-value in place of *α*. Because Pr(*P* ≤ *α*|*H*_*A*_) ≥ Pr(*P* ≤ *α*|*H*_0_), the result can never be larger than the prior probability, Pr(*H*_0_). For the result to be interpreted as the probability of no true association between a genetic variant and disease given a P-value, a modification is needed, as described in the next section.

### 4.2 POFIG: Probability that a finding a genuine

In this section, we present POFIG via its relation to FPRP, assuming the point null hypothesis, i.e., exactly zero effect size for false signals. To obtain the posterior probability of *H*_0_ given a particular P-value, one cannot simply substitute *α* in Eq (12) for the P-value (*p*), because the ratio of CDFs, $F_{\bar{\gamma }}(p)/F_{0}(p)=F_{\bar{\gamma }}(p)/p$ would refer to the entire interval [0, *p*]. To assess the posterior probability of *H*_0_ given an observed P-value, one could consider a narrow interval around *p* instead. This is simply achieved by replacing the ratio of CDFs by the ratio of densities:

$$\begin{array}{ccc} \mathrm{Pr}(H_{0}|p) & = & \lim_{\delta \to 0}\mathrm{Pr}(H_{0}|P\in \left[p,p+\delta \right])\\ & = & {\left[1+\frac{1-\mathrm{Pr}(H_{0})}{\mathrm{Pr}(H_{0})}\times \frac{f_{\bar{\gamma }}\left(p\right)}{f_{0}\left(p\right)}\right]}^{-1} \end{array}$$

As in the original FPRP approach, we will assume that P-values follow the uniform distribution under *H*_0_, i.e., *f*_0_(*p*) = 1, so that the probability that the finding is genuine is

$$\begin{array}{c} \text{POFIG}=\mathrm{Pr}\left(H_{A}|p\right)=1-\mathrm{Pr}\left(H_{0}|p\right)=1-{\left[1+\frac{1-\mathrm{Pr}(H_{0})}{\mathrm{Pr}(H_{0})}\times f_{\bar{\gamma }}\left(p\right)\right]}^{-1} \end{array}$$ (13)

where $f_{\bar{\gamma }}(p)$ is the marginal P-value density that acknowledges that different signals have different effect sizes. The next subsection presents basic ways to take this distribution into account in computing the averaged (“marginal”) P-value PDF. Conceptually, 1-POFIG corresponds to the local FDR [33, 34]. The equivalence of local FDR and the “point null” version of POFIG is in that they are both inversions of the conditional probability. Both can be described as an application of the Bayes rule. There are several differences between the approaches. One difference is that P-value and POFIG ranks are not necessarily the same, while local FDR (and q-values) have the same ranks as P-values. Further, POFIG in its general form, where the null hypothesis is not a point, Eq 3) is no longer equivalent to the local FDR. We argue that this form is a more biologically realistic representation of the effect size distribution; not only the point null is a statistical abstraction, but it is expected that most genetic variants with heritable contribution have only tiny effect on a trait. Another distinction is in how the P-value distribution is modeled. Local FDR relies on an Empirical Bayes approach which uses the totality of data to evaluate prior parameters. This approach works well when non-null signal frequency is high, as found in differential gene expression studies. In contrast, q-value and local FDR approaches are not commonly applied in genetic association studies, where they may not be reliable due to high sparsity of true signals [35].

#### 4.2.1 Marginal P-value density: Continuous approach

1. If the sample size is the same or similar for all tests, as can be found in SNP association studies, one can specify the distribution for the parameter *γ* directly: *γ* ∼ Γ(·). Realistic shapes for the effect size distribution in association studies would assume that there are many small effects and few that are large. Effect size distributions for such genetic association signals follow an “L-shape” that is well approximated by a Gamma distribution with the shape parameter smaller or equal to one [16]. There are some advantages to this approach. As suggested by Park et al.[7], when *γ* is defined as in Eq (6), then *γ* × 2/*N* can serve as a definition of the effect size and corresponds to the contribution of a SNP to the additive genetic variance of the trait. It is also very general in that *γ* may refer to a parameter that is measuring a deviation from *H*_0_ for a variety of test statistics, including noncentrality for chi-square and F distributions, and mean shift for normally or t-distributed test statistics. It has been noted that because *γ* values would typically depend on allele frequency, an implicit assumption in modeling the distribution of *γ* directly is that less frequent alleles tend to have larger effect size [36]. With this approach, the marginal P-value PDF is
  $$\begin{array}{ccc} f_{\bar{\gamma }}\left(p\right) & = & \int f_{\gamma }\left(p|\gamma \right)\Gamma \left(\gamma \right)\;d\gamma \end{array}$$ (14)
2. When sample sizes (*N*) differ among tests, we can take advantage of the fact that *N* often appear as a separate term (as in Eq 6) and specify a distribution for the standardized effect size, ℰ ∼ Γ_ℰ_(·). Contingency table chi-square noncentrality can be similarly written in terms of the actual (*o*_*i*_) and expected *e*_*i*_ frequencies as *γ* = *N* × ∑(*o*_*i*_ − *e*_*i*_)^2^/*e*_*i*_. For the normal test statistics, $\sqrt{N}$ factors out instead. Thus, allowing for varying sample sizes of different tests, the marginal P-value PDF is
  $$\begin{array}{ccc} f_{\bar{\gamma }}\left(p\right) & = & \frac{1}{\sigma }\int f_{\gamma }(p|\gamma )\Gamma_{𝓔}(\frac{\gamma }{\sigma })\;d\gamma \end{array}$$ (15)
  where *σ* is equal to *N* for chi-square and $\sqrt{N}$ for normal test statistics.
3. It is also possible to specify a distribution for a non-standardized effect size, defined, for example, by the square of log odds ratio, *D*, as in the logistic model (Eq 6). This model specifies that *D* follows the distribution Γ_*D*_(·). In this case, the marginal PDF is a function of both the P-value and the allele frequency, *q*. Then the marginal P-value PDF is
  $$\begin{array}{ccc} f_{\bar{\gamma }}\left(p\right) & = & \frac{1}{\sigma }\int f_{\gamma }(p|\gamma )\Gamma_{D}(\frac{\gamma }{\sigma })\;d\gamma \end{array}$$ (16)
  In this specification, $\sigma =N\hat{q}(1-\hat{q})$, where $\hat{q}$ is the sample pooled allele frequency.

Given the three alternative specifications of the marginal P-value density just described, we will consider two approximations that do not require the complete specification of the effect size distribution, Γ(·) and allow to sidestep calculation of the integral.

#### 4.2.2 Marginal P-value density: Typical effect size method

The first approximation is reminiscent of the FPRP approach where a “typical” single value is considered in place of the entire effect size distribution. By writing Eq (14) as an expectation and applying the first order Taylor expansion, we obtain an approximation as

$$\begin{array}{ccc} f_{\gamma }\left(p\right) & = & E\{f_{\gamma }\left(p|\gamma \right)\}\\ & \approx & f_{\gamma }\left(p|\mu_{\gamma }\right) \end{array}$$ (17)

where *μ*_*γ*_ is the mean value of the effect size distribution. With the specification by Eq (14), *μ*_*γ*_ = *E*(*γ*). With the specifications by Eqs (15, 16), *μ*_*γ*_ = *σ* × *E*(ℰ) and *μ*_*γ*_ = *σ* × *E*(*D*), respectively. This approximation is appealing primarily because it is a much simpler task for a researcher to specify a single “typical” value than to come up with the entire effect size distribution. Here, we consider utilization of the mean primarily as a way of misspecification of the effect size distribution. The effect size distribution can be misspecified in an infinite number of ways, and reducing the distribution to just the mean gives one such natural way. The effect of misstating the mean would introduce further bias in posterior estimates, however it is difficult to systematically evaluate that bias, since its degree would depend on many parameters, including sample size. Nevertheless, for any specific study, researchers may evaluate the effect of varying the mean on robustness of the posterior inference.

#### 4.2.3 Marginal P-value density: Tabulated values (discretization) method

While the simple “mean” approximation can be sufficient, sometimes it may introduce a noticeable imprecision. The second approximation is based on discretizing the effect size distribution into several “bins”. While only slightly more complex than the previous approximation, our results indicate that usage of as little as three bins provides sufficient precision. One possible way, based on the extension of the previous single-value method is to take the middle bin to be centered around the mean value of the effect size distribution *μ*, with the left and the right sides extending to *μ* − *μ*/2 and *μ* + *μ*/2, respectively. Assuming the majority of signals are of small size and signals of large magnitude are rare, the effect size distribution can be represented by a Gamma(shape, scale) distribution with the shape smaller or equal to one. These distributions taper off as shown in Fig 1 and the mean value is *μ* = scale × shape. The figure also illustrates a representation of a continuous distribution by three mean values for each of three bins. Each bin is characterized by its mean effect size value *μ*_*i*_ and the relative proportion (signal count) for the bin, *w*_*i*_. The marginal density approximation is

$$\begin{array}{ccc} f_{\bar{\gamma }}\left(p\right) & \approx & \frac{\sum_{i=1}^{B}w_{i}f_{\gamma }\left(p|\gamma_{i}\right)}{\sum_{i=1}^{B}w_{i}} \end{array}$$ (18)

where *B* is the number of bins. With the specification given by Eq (14), *γ*_*i*_ = *μ*_*i*_. With the specifications given by Eqs (15, 16), *γ*_*i*_ = *μ*_*i*_ *σ*. Park et al. designed a method for tabulating effect sizes with their respective abundances (*w*_*i*_) based on GWAS data and provided such tabulated values for several diseases. In their definition, effect sizes are expressed as *γ* × 2/*N* (where *γ* and *N* are given by Eq 6).

### 4.3 Simulation experiments

Each simulation experiment consisted of *K* one degree of freedom chi-square allelic association tests. Each test with its corresponding P-value was randomly chosen to represent either a genuine or a spurious signal with respective probabilities 1 − Pr(*H*_0_) for genuine and Pr(*H*_0_) for spurious signals. For experiments with *K* ≤ 10000 tests, we assumed two distinct proportions of false signals. Scenario 1 was modeled after a “fishing expedition”, using a relatively large proportion 0.9. Scenario 2 was “we know what we are doing” setup with the smaller proportion of 0.5. For simulations with a very large number of tests, *K* = 10^6^, we used much larger proportions of false signals, 1-(150/10^6^) and 1-(1000/10^6^) that are expected in genome wide association studies. These proportions and the number of tests are given in Table 1.

For a given genuine signal, its effect size *D* was sampled from a gamma distribution with parameters as will be specified below. Then, the population SNP frequency *q* was sampled from the uniform (0.05, 0.95) distribution. For a given sample size parameter *N* (given by Eq 6), the noncentrality parameter was determined as *γ* = *N* *D* *q*(1 − *q*), according to Eq (6). Next, the P-value for this particular genuine effect was sampled as follows: (1) sample a normal Z-score *Z* from the standard normal distribution; (2) convert *Z* to a noncentral chi-square statistic $X={\left(Z+\sqrt{\gamma }\right)}^{2}$; (3) calculate the P-value using the central chi-square cumulative distribution function (CDF) with one degree of freedom, *G*_0_(·), as P-value = 1 − *G*_0_(*X*). The value *γ* was discarded, i.e., it was assumed to be unknown, and only the P-value was stored. P-values for spurious signals were sampled from the uniform (0,1) distribution.

For the effect size distribution, we assumed an L-shaped gamma distribution with the shape equal to one. We assumed the mean odds ratio of 1.1 from which the effect size distribution is given approximately by Gamma(1, [log(1.1)]^2^). Sample sizes are given in footnotes for the tables of results (Tables 1, 2 and 3). Since effect sizes were modeled in terms of odds ratios instead of the noncentralities, the marginal P-value density for the exact method was computed via Eq (16). Allele frequency was binomially sampled given the signal-specific population allele frequency and the sample size. The noncentrality parameter was computed via Eq (6). Eq (1) was used to obtain the posterior probability, assuming known prior proportion of spurious signals. Note that the method of modeling the effect size distribution in terms of odds ratios, applied in these simulations does not preserve the ranking of P-values when they are converted to the posterior probability of *H*_0_. This is because two SNPs with the identical odds ratios may have different allele frequencies, and the allele frequency is a part of the posterior computation.

#### 4.3.1 Scan, replication and combined probabilities that the finding is genuine

To illustrate the distribution of scan, replication and combined posterior probabilities as well as their accuracy in distinguishing between genuine and spurious signals, we used a simulation setup similar to the one just described. In each of these experiments, we took the smallest P-value (minP) from the scan (*N* = 4000), and generated a random replication P-value, re-using the effect size associated with the scan minP. For the replication study, we assumed a four times larger sample size, Pr(*H*_0_) = 0.99, and the number of tests equal to *K* = 10000. Probabilities for the replication samples were computed in a conservative way, using the same effect size distribution as that assumed for the scan, although the effect size distribution in replication studies is expected to be modified.

One can most easily combine scan and replication posterior probabilities, or more generally, probabilities from *S* independent studies if P-values are reported as one-sided, that is, when a specific direction of association is being tested. In this case, the distribution of effect sizes needs to reflect the effect direction as well, for example, log odds ratios instead of their squared values might be used. Then Eq (1) is modified as

$$\begin{array}{c} \text{POFIG}=1-{\left[1+\frac{1-\mathrm{Pr}(H_{0})}{\mathrm{Pr}(H_{0})}\times \prod_{j=1}^{S}f_{\bar{\gamma }}\left(p_{j}\right)\right]}^{-1} \end{array}$$ (19)

Most association P-values are reported as two-sided, i.e., based on an association statistic where the direction of association is not reflected. There is also convenience in using two-sided P-values, in which case the effect size distribution is specified based on its magnitude, without taking into account the sign of the effect. A simple way of taking the effect sign into account is to convert two-sided scan and replication P-values to one-sided, then combine one-sided P-values, and convert the result (*p*_o_) back to a two-sided P-value, *p*_t_. The resulted combined P-value approximates well the P-value that would have been obtained by pooling individual-level data [37]. In general, several two-sided P-values (*p*_*i*_, *i* = 1, …, *k*) can be combined with this method as follows:

$$\begin{array}{ccc} p_{\text{o}} & = & 1-\Phi [\frac{\sum_{i=1}^{k}[2I({\text{sign}}_{i}={\text{sign}}_{1})-1]w_{i}\Phi^{-1}\left(p_{i}/2\right)}{\sum_{i=1}^{k}w_{i}^{2}}] \end{array}$$ (20)

$$\begin{array}{ccc} p_{\text{t}} & = & 2\min(p_{\text{o}},1-p_{\text{o}}) \end{array}$$ (21)

where *I*(·) is the indicator function, sign_*i*_ is the effect direction associated with the P-value *p*_*i*_, Φ^−1^(·) is the inverse normal CDF, and *w*_*i*_ are the weights which can be taken to be square roots of the sample sizes (square root of the harmonic mean of case and control sample sizes for case-control studies). Such combined P-value can be converted to the posterior probability in the same way as the original P-values (using Eq 1), but utilizing the total sample size of the studies. We illustrated this approach via simulation experiments (as summarized in Fig 2).

### 4.4 POFIG application to Crohn’s disease P-values

For Crohn’s disease, the total number of susceptibility loci was estimated together with the tabulated effect size distribution by Park et al.[7] and given in their supplementary Tables 3 and 5 in a standardized fashion as 2*Dq*(1 − *q*) which is 2/*N* of the noncentrality for the logistic model (Eq 6). Therefore, we modeled the marginal P-value density, which is a part of Eq (1) for the posterior probability by assuming the distribution for the noncentralities directly (Eq 14). This method preserves the ranking of P-values when they are converted to the posterior probability of *H*_0_. We assumed a gamma distribution with the shape parameter equal to 1. The second parameter of the gamma distribution, its scale, was obtained using its property that mean = shape × scale. Park’s Supplementary Table 5 was used to derive the mean effect size value. We used Park’s estimate of 142 susceptibility loci. The “loci” in this case correspond to associated regions, defined by Barrett et al. on the basis of linkage disequilibrium, with the average locus length equal to 232973 base pairs. Taking the total genome length to be 3 billion base pairs, the number of segments with that length in the genome is 12877. We used these numbers to derive the prior for the *H*_0_ as 1 − 142/12877. Additionally, we evaluated our POFIG approach with different priors and effect size distributions. We considered different estimated numbers of susceptibility loci obtained by different methods as given in Supplementary Table 5 of Park et al. For the approach that assumes a continuous effect size distribution, we utilized a gamma distribution with the shape parameter equal to 1 or 0.634. The value 0.634 for shape parameter value was chosen to yield the median value of the noncentrality distribution to be twice as small as that of the distribution with the shape equal to 1 for the same value of the scale. Again, the second (scale) parameter was determined by the mean effect size value.
